# Antioxidant and Anti-Inflammatory Properties of Melatonin in Secondary Traumatic Brain Injury

**DOI:** 10.3390/antiox14010025

**Published:** 2024-12-28

**Authors:** Mariusz Sieminski, Michalina Reimus, Maria Kałas, Ewelina Stępniewska

**Affiliations:** 1 Department of Emergency Medicine, Medical University of Gdańsk, 80-214 Gdańsk, Poland; marysia88@gumed.edu.pl (M.K.); e.stepniewska@gumed.edu.pl (E.S.); 2 Emergency Department, University Clinical Center, 80-952 Gdańsk, Poland; mreimus@uck.gda.pl

**Keywords:** melatonin, traumatic brain injury, oxidative stress, neuroinflammation

## Abstract

Traumatic brain injury (TBI) is a disease resulting from external physical forces acting against the head, leading to transient or chronic damage to brain tissue. Primary brain injury is an immediate and, therefore, rather irreversible effect of trauma, while secondary brain injury results from a complex cascade of pathological processes, among which oxidative stress and neuroinflammation are the most prominent. As TBI is a significant cause of mortality and chronic disability, with high social costs all over the world, any form of therapy that may mitigate trauma-evoked brain damage is desirable. Melatonin, a sleep–wake-cycle-regulating neurohormone, exerts strong antioxidant and anti-inflammatory effects and is well tolerated when used as a drug. Due to these properties, it is very reasonable to consider melatonin as a potential therapeutic molecule for TBI treatment. This review summarizes data from in vitro studies, animal models, and clinical trials that focus on the usage of melatonin in TBI.

## 1. Introduction

The term “traumatic brain injury” (TBI) is defined as a situation where external physical forces acting against the head (or skull) lead to temporary or chronic damage to the brain [1]. The damage may be of a functional nature—manifesting solely as a transient loss of consciousness resulting from the short-lasting dysfunctio of neurons of the reticular formation, without further sequelae—or of a structural nature, associated with the destruction of neural cells and fibers, which may happen in traumatic intracerebral hematoma. Classically, TBIs can be classified as mild, moderate, or severe based on a patient’s Glasgow Coma Score during admission [2]. It must be remembered that the term “mild” can be misleading, as any trauma-related brain injury may cause debilitating and life-long consequences. Another common mistake is the opinion that TBI is a transient incident and that a clinical issue is resolved when a negative head computed tomography result is obtained. Meanwhile, even “mild” TBI may trigger potentially devastating oxidative and inflammatory processes, resulting in significant damage to neural tissue.

### 1.1. Epidemiology of TBI

TBI is one of multiple “silent epidemics” affecting our civilization. In contrast to other civilizational health challenges, like dementia or cardiovascular diseases, victims of TBI are often young and professionally active, which makes TBI-related mortality and disability an important social problem.

The overall incidence of TBI in Europe, according to the most recent review of the literature, is 258 cases per 100,000 inhabitants [3]. This estimation should be treated with caution, as the authors used data sourced from different countries, regions, and timespans. Globally, it is estimated that 50,000,000–60,000,000 patients experience TBIs every year [4]. Data from other continents reveal a higher prevalence of TBI. The Global Burden of Diseases report indicated that the global, age-standardized incidence of TBI was 369 cases per 100,000 inhabitants in 2016 [5]. We have data revealing that the incidence of concussion (which may be regarded as a form of mild TBI) reaches 1153/100,000 inhabitants [6]. It must be remembered that most TBI cases are mild, with the percentage of mild TBIs ranging from 71% to 97.5% (such a wide span resulted from the different criteria that were used) [7].

The incidence of TBI varies between countries, reaching higher values in low- and middle-income countries [8]. Moreover, the distribution of the mechanisms of TBI varies between countries, with road traffic accidents being the most significant cause of TBI in low- and middle-income countries, while falls are the most common cause in high-income countries [3,5,8]. The fact that falls are the main cause of TBI is related to the fact that there is an overrepresentation of elderly patients among the victims of TBI. They constitute approximately 30% of all TBI cases, 43.9% of patients hospitalized due to TBI, and 38.4% of TBI-related mortality [4,9].

TBI is also one of the leading causes of death, especially in the population of young adults. The TBI-related mortality rate in Europe ranges from 9 to 28.1 per 100,000 citizens, with approximately 82,000 Europeans dying from TBI and its acute consequences each year [3,4]. This high mortality is one of the reasons why TBI is such a social burden, as it translates to 160 years of life lost (YLLs) per 100,000 inhabitants in the European Community. This burden also expands to 8.1 million years lived with disability globally [4,5]. The global direct (medical) cost of TBI in 2016 in the USA was USD 40.6 billion, with mild TBIs causing most of the costs [10].

### 1.2. Aim of the Review

As presented above, TBI is a medical challenge for our civilization that causes too many lives and years of life to be lost. The long-term consequences of TBI, like post-traumatic headaches, cognitive disorders, and post-traumatic epilepsy, are significant burdens to healthcare systems. Therefore, any therapy with the potential to stop the trauma-evoked cascade of brain damage is worthy of consideration. The most potent mechanisms of secondary (and, therefore, potentially preventable) brain injury are oxidative stress and neuroinflammation. As TBI damages cellular membranes and increases their permeability, ions lead to mitochondrial dysfunction and massive production of reactive oxygen and nitrogen species, which cause a vicious cycle of membrane destruction and cell death. This process develops and is exacerbated in inflammatory environments created by excessively produced cytokines and activated immunocompetent cells. Melatonin, with its known antioxidant potential and ability to inhibit the inflammatory process, is a potential treatment for TBI. This review aims to present the antioxidative and anti-inflammatory potential of melatonin in the context of the mechanisms of TBI.

## 2. Mechanisms of TBI

### 2.1. Mechanisms of Primary TBI

TBI can be described, in the simplest terms, as the result of an external force acting on the human head. This force can originate from various sources: a blunt impact, a penetrating missile, rapid acceleration or deceleration during a car accident (even in the absence of physical contact), rotational forces, or a shockwave from a nearby explosion. For brain injury to occur, the energy from the impact must be transferred through the outer layer of the skull. It is incorrect to think of the skull as a uniform sphere. In reality, it is composed of four pairs of bones (frontal, parietal, temporal, and occipital), which differ significantly in their shapes and mechanical properties. For instance, the concave temporal bone absorbs more energy from a direct impact compared to the convex parietal bone [11]. These bones also vary in thickness and in the proportion of spongy versus compact bone, with spongy bone being more capable of absorbing energy due to its structure [12]. This means that the amount of energy transferred to the cranial cavity depends not only on the initial energy of the impact but also on the anatomical site of the trauma.

The most significant consequence of this energy transfer is the acceleration imparted to the contents of the skull. These contents are typically divided into three components: blood circulating through the cerebral vasculature, cerebrospinal fluid, and the brain. The brain itself is not a homogeneous structure; different regions of the brain exhibit distinct mechanical properties depending on their composition (e.g., white matter versus gray matter), cellular types, and densities [13]. As a result, the same force acting on a particular brain region will impart different accelerations to specific structures. For example, the cerebral cortex will accelerate differently from the subcortical white matter. This differential motion creates shearing forces at the interfaces between gray and white matter, potentially leading to axonal injury or concussion. When these shearing forces act on both brain tissue and blood vessels, vessel walls can tear, causing subdural or intracerebral hemorrhage.

Similar mechanical differences exist at the cellular and subcellular levels. An axon subjected to linear or rotational acceleration will respond differently than the soma of a neuron. Shearing or tearing an axon disrupts the cytoskeleton and impairs intracellular transport [14]. Mechanical damage to the cell membrane leads to the release of pro-inflammatory cytokines. Additionally, mechanical stress affects the function of transmembrane ion channels, resulting in a significant influx of calcium and sodium ions. This triggers the uncontrolled release of neurotransmitters and initiates processes leading to cell death. Disruption of the cell membrane further depletes ATP and impairs mitochondrial function, which can promote apoptosis [15].

In summary, primary TBI caused by external forces leads to the mechanical disruption of brain tissue, primarily due to its mechanical heterogeneity. This can result in axonal injuries, concussions, contusions, and hemorrhages. At the cellular level, forces acting on neurons damage the neurolemma (particularly in axons), leading to metabolic dysfunction, the uncontrolled release of neurotransmitters and cytokines (which contribute to secondary brain injury), and apoptosis.

Although many events happen simultaneously during the pathological cascade of TBI, the following chronology may be imagined: the primary physical impact on neural cells (described above), the release of abnormal quantities of neurotransmitters, an increase in blood–brain barrier (BBB) permeability and an influx of pro-inflammatory cytokines and cells, and the development of neuroinflammation with simultaneous dysfunction of cerebral vascular autoregulation.

### 2.2. Dysfunction of Neurotransmission

Stretching and tearing forces acting on neural cells cause changes in the properties of transmembrane ion channels, leading to their uncontrolled opening and a significant influx of calcium ions into the cells [15]. An increase in the intracellular concentration of calcium provokes increased release of glutamate [16]. Simultaneously, trauma to neural tissue leads to a significant decrease in the synaptic re-uptake of glutamate [17]. The consequence of these two phenomena is a considerable increase in the intracellular concentration of calcium ions, which on the one hand promotes a further increase in the release of glutamate (closing a positive feedback loop), and on the other activates numerous catabolic enzymes (e.g., proteases and phospholipases) that destroy cellular membranes and organelles (e.g., mitochondria), finally leading to apoptosis. Glutamate-related calcium influx dysregulates mitochondrial processes and evokes oxidative stress and the production of reactive oxygen and nitrogen species.

Apart from an increase in glutamatergic transmission, traumatized neurons start to rapidly metabolize catecholamines (e.g., noradrenaline), with parallel downregulation of their receptors. This leads to a generalized drop in catecholaminergic stimulation, which has detrimental impacts on the level of consciousness (in the acute phase) and on concentration and learning abilities (in the chronic phase) [15,18]. Similar changes in dopaminergic transmission have been described, with sudden abundant release immediately after trauma and a chronic dopaminergic deficit (resulting from a disturbed rhythm of dopamine turnover), which has the clinical effects of mood and cognitive disorders in the chronic phase of TBI [19].

Studies on the secretion of melatonin in humans after TBI have produced conflicting results. Some authors have observed reduced melatonin levels in post-traumatic patients, which have often been accompanied by disturbances in the sleep–wake cycle [20,21]. However, other studies have reported sleep disorders in TBI patients without finding decreased melatonin levels [22].

In summary, TBI-evoked disturbances in neurotransmission lead to calcium-dependent cellular death in the acute phase of TBI, along with chronic disorders of arousal and cognition.

### 2.3. Blood-Brain Barrier Dysfunction in TBI

Immediately after brain trauma, a collapse of the blood–brain barrier (BBB) is observed, with an increase in its permeability. This results from the destruction of tight junctions between endothelial cells. Tight junctions, which control the flow of ions, molecules, and cells through the BBB, are constructed with specific proteins: occludins, claudins, and ZO-1. It was shown that trauma causes significant reductions in the expression of these proteins, automatically leading to an increase in the permeability of the BBB [23]. It was shown that the first phenomenon following trauma is a loss of pericytes, which form the base of the BBB with endothelial cells. The loss of these cells is a consequence of the downregulation of platelet-derived growth factor (PDGF) signaling. A reduction in the expression of proteins that form tight junctions is observed after the loss of pericytes [24]. All of these changes lead to an increase in the permeability of the BBB, with heightened presence and activity of aquaporin 4, more intense inflow of water into the central nervous system, and development of brain edema [25].

Apart from water influx, the increased permeability of the BBB allows for an influx of pro-inflammatory cytokines and cells of the immune system, such as neutrophils and macrophages, which intensify the inflammatory trauma response initiated by immunocompetent cells within the central nervous system. Therefore, an impaired BBB contributes to an increase in brain edema and a sustained inflammatory response to trauma. Taking into consideration the fact that tight junction dysfunction may last for years after trauma, it becomes evident that BBB impairment is partially responsible for both the acute (e.g., edema) and chronic consequences of TBI [26,27].

### 2.4. Oxidative Stress in TBI

TBI-related injuries in neural cells, primarily resulting from increased glutamatergic neurotransmission and changes in the permeability of transmembrane ion channels, with high influxes of calcium ions into the cells, lead to severe metabolic dysfunction with increased production of free radicals. Changes in the calcium concentration cause increased permeability of mitochondrial membranes and influxes of ions into mitochondria. The presence of excessive amounts of calcium ions within the mitochondria renders the electron transport chain of the inner mitochondrial membrane ineffective, and free electrons that appear rapidly join oxygen atoms. This generates free radicals. Among them, hydrogen peroxide, superoxide anions, and peroxyl radicals are the most common [28,29]. Simultaneously, an ineffective electron transport chain leads to the generation of reactive nitrogen species. Nitrosative stress is another phenomenon that damages neural tissue in parallel with oxidative stress, as described by Reed et al. [30]. Neural tissue is rich in lipid acids, which serve as the primary structural components of the neurolemma and myelin. These acids are highly susceptible to attacks by free radicals, so the situation described above, combined with mechanical injuries to cellular membranes, leads to massive damage to membranes and cellular death. Damaged membranes release significant amounts of arachidonic acid, which rapidly reacts with free radicals and is oxygenated to form hydroperoxide radicals, which further increase the imbalance between the total concentration of reactive oxygen species and the antioxidant potential of the neural tissue, deepening the oxidative stress [31,32]. All of these processes take place in an environment containing massive amounts of catecholamines, which were released immediately after the trauma. It must be noted that catecholamines are very prone to oxygenation (especially in conditions of oxidative stress), so they become another source of free radicals that attack cellular membranes [33]. Simultaneously, activation of cerebral immunocompetent cells (glial cells) and migration of immune cells (mostly neutrophils) towards the brain take place. These cells produce reactive oxygen species, mainly through the activity of NADPH oxidase 2 (NOX2) [34]. Therefore, the uncoupled electron transport chain, oxygenation of arachidonic acid, oxygenation of catecholamines, and production of ROS by immune cells constitute elements of a closed positive feedback loop leading to massive production of free radicals and an increase in oxidative stress. Brain tissue is characterized by limited antioxidant activity. Therefore, it is defenseless against oxidative damage to cellular membranes, leading to widely distributed apoptotic processes. Neuroprotective interventions for this condition require external sources of antioxidant activity provided by therapeutic substances with reductive potential, such as melatonin.

### 2.5. Neuroinflammation in TBI

An inflammatory reaction is a physiological defense against any condition threatening the homeostatic stability of a tissue or an entire organism. Certainly, TBI is a condition that threatens stability. Therefore, it evokes an inflammatory response that exerts a neuroprotective effect and influences tissue repair but also has negative effects, leading to further tissue damage. During the acute phase of TBI, the latter effects are more abundant and should be inhibited before they transform into chronic neuroinflammation.

The first line of immunological defense within the brain is constituted by microglia and astrocytes. Reacting to mechanical stimulation and damage to neural cells in the acute phase of TBI, these cells enter an activated state. They undergo a morphological change and start to increase the release of cytokines (Il-1, Il-6, and TNF-alpha), chemokines, and matrix metalloproteinases (MMP-9) [35,36]. The most clinically visible effect of this activity is increased expression of the membrane water channel aquaporin 4, leading to increased permeability of neural membranes, an influx of water into cells, and cytotoxic brain edema [37]. Pro-inflammatory cytokines stimulate the expression of the NLRP3 inflammasome in neural cells, promoting a pro-apoptotic state that ultimately results in pyroptotic cell death [38]. Another consequence of glial release of cytokines is depressed expression of proteins that build tight junctions within the BBB (e.g., claudins and occludins), which results in a loss of BBB tightness and increased influx of peripheral cells (especially immune cells) into the central nervous system [39]. Activated microglial cells are also (as mentioned earlier) active producers of reactive oxygen species, leading to an intensification of oxidative stress.

High concentrations of chemokines and metalloproteinases create an environment that encourages peripheral immune cells to infiltrate the injured brain through the damaged BBB [40]. The first cells to respond are neutrophils, followed by monocytes that transform into macrophages. Their presence escalates the inflammatory response, which has positive effects (e.g., cleansing cellular debris and protection against infectious factors) as well as negative ones. Migrated immune cells enhance the production of free radicals and cytokines, which, in turn, intensify damage to cellular structures and pyroptosis. Another consequence of inflammatory cellular activity (both microglial and neutrophil-derived) is activation of the complement system and subsequent formation of a membrane-attacking complex [41,42]. The latter destroys the membranes of cells in neural tissue, leading to their deaths. As described earlier, a constant element of TBI-related changes is increased permeability of the blood–brain barrier. This allows antigens from destroyed neural cells to enter the systematic circulation and contact antigen-presenting cells (APCs) [43]. APCs recognize neural antigens as pathological ones and activate B cells, which start to produce anti-neural antibodies. The latter flow into the cerebral environment and connect with specific antigens, triggering an autoimmunological reaction that directly damages the cells and increases phagocytosis. This may provoke ongoing neuroinflammation and constant production of anti-neural antibodies [44].

Based on the above description, the inflammatory cascade provoked by TBI may lead to secondary brain injury in multiple ways unless it is limited by innate anti-inflammatory mechanisms or external interventions with molecules characterized by anti-inflammatory potential, such as melatonin.

### 2.6. TBI-Evoked Vascular Disturbances

The brain is an organ that is highly dependent on a continuous supply of glucose and oxygen, and even a very short decrease in blood flow may lead to an injury in the neural tissue. Stable cerebral blood flow is ensured through a physiological mechanism called cerebrovascular autoregulation. This autoregulation is an effect of a complex and constantly ongoing interplay of vasogenic and metabolic factors, resulting in precise adjustments of cerebral vessel diameters based on actual metabolic needs, systemic blood pressure, and intracranial pressure [26]. TBI due to increased catecholaminergic transmission and changes in intracellular ion concentrations (influx of sodium and calcium ions forcing increased activity of ATPase) is a condition with elevated energetic needs and higher demand for oxygen and glucose, which in fact means that more intense cerebral perfusion is needed. This implies a shift in cerebrovascular autoregulation towards dilatation of cerebral vessels. Meanwhile, TBI provokes a series of processes that act against proper regulation of the diameters of cerebral vessels. Brain injury causes rapid activation of the sympathetic system, with a marked tendency toward vasoconstriction in parallel with tachycardia and an increase in blood pressure that challenges the autoregulation system [45,46,47]. Activation of the sympathetic system and the renin–angiotensin–aldosterone axis leads to secretion of endothelin-1, which counteracts automated cerebral vasodilation [48]. This simultaneous process is increased, followed by a decrease in the expression of contractor proteins in the cells of the smooth muscles within the walls of arteries [49,50]. Moreover, in some studies, reductions in the vasculatures of neural tissues were described [51].

In summary, although TBI requires increased cerebral blood flow, a series of events following trauma inhibit cerebrovascular autoregulation, deepening the injury to the neural tissue.

## 3. Antioxidant and Anti-Inflammatory Properties of Melatonin

### 3.1. Physiological Role of Melatonin

Melatonin is a neurohormone that is primarily synthesized in the pineal gland. It is a derivative of tryptophan, specifically N-acetyl-5-methoxytryptamine. In addition to the pineal gland, melatonin is produced in the retinas and by cells of the immune system [52]. It is produced and released in a circadian rhythm, with the lowest concentrations observed during the day (<2 pg/mL) and the highest at night (approximately 100 pg/mL) [53]. Research has shown that, under certain conditions, local tissue concentrations of melatonin may be significantly higher than those in plasma [54]. Melatonin acts through two G-protein-coupled receptors, MT1 and MT2, which are most abundantly found in the pars tuberalis of the pineal gland, retinas, and hypothalamus [55].

The primary physiological role of melatonin is the regulation of circadian rhythms; it switches the body to “sleep mode”. Melatonin’s production and secretion are inhibited by light and increased in darkness [56,57]. The locations of melatonin receptors suggest that this neurohormone modulates the functions of neural and neuroendocrine centers and directly affects various systems and organs [52,56]. Melatonin’s chronobiological role involves driving the rhythmic functions of peripheral tissues and organs, aligning them with the day–night cycle [58].

As the transition to “sleep mode” involves modulating the autonomic nervous system, it has been shown that melatonin can reduce sympathetic activity [59,60]. By reducing vasoconstriction, melatonin lowers blood pressure, making it a potential adjunct treatment for hypertension [61]. Furthermore, melatonin significantly affects metabolism, serving as a protective factor against obesity and diabetes mellitus [62,63,64]. Melatonin also modulates the inflammatory response; it possesses notable anti-inflammatory properties that can prevent cell death caused by pyroptosis [65,66].

### 3.2. Antioxidant Properties of Melatonin

The destruction of neural cells, pathological activity of neurotransmitters, dysfunction of mitochondria, and inflammatory reactions resulting from TBI lead to oxidative stress and increased production of free radicals. These pathological phenomena damage proteins, phospholipids of cellular membranes, and DNA, which may, in turn, trigger uncontrolled apoptotic processes and, ultimately, lead to the death of cells. This lethal cascade may be inhibited by the actions of antioxidants (which are capable of reducing the production of free radicals) and scavengers (which directly trap free radicals) [52].

Melatonin has been studied extensively as an antioxidant therapeutic factor. Data collected thus far show that it may act protectively at every step of oxidative damage in tissues: it prevents the production of oxidants, scavenges free radicals, and repairs damaged molecules. The production of oxidative molecules requires metal ions, such as iron (Fe) or copper (Cu). Thus, chelation of these ions suppresses the release of free radicals. Melatonin reacts with copper and iron, trapping these ions in complexes and eliminating them from the intra- and extracellular environments. This, in turn, inhibits the metal-dependent production of free radicals [67,68,69]. Chelation of ferric ions using melatonin has been proven to inhibit Fe-dependent cellular death (ferroptosis). Gas et al., in a mouse model of TBI, observed that exogenous melatonin prevented neurological deterioration by limiting the ferroptotic process [70]. Similar results were obtained in an animal TBI model by Rui et al. Treatment with melatonin lowered the number of iron-positive cells, improved behavioral scores, and reduced neural injury [71]. Apart from limiting the production of oxidants, melatonin can also scavenge them. A direct reaction of melatonin with a hydroxyl radical was described [72,73], as was its participation in the elimination of peroxyl radicals and nitric oxide [74,75,76]. Melatonin and its metabolites are capable of scavenging reactive nitrogen compounds. A further melatonin-related reduction in the load of free radicals is a result of its ability to activate enzymatic pathways for the elimination of oxidants. Melatonin may activate glutathione peroxidase and other antioxidant enzymes [77,78]. One of the crucial features of melatonin is its ability to activate Nrf2 (nuclear factor erythroid 2-related factor 2), which, in turn, upregulates the expression of genes of antioxidant enzymes. It was proven that this mechanism exerts a neuroprotective effect in ischemic brain injury [79,80]. The antioxidant potential of melatonin is additionally potentiated by the fact that its metabolites (e.g., methoksykynuramines and hydroxymelatonins) exert similar (chelating and scavenging) effects [81,82]. The final stage of the antioxidant action of melatonin is its potential to repair molecular damage caused by free radicals. It has been proven in various animal models (e.g., models of ischemic or traumatic injuries in neural tissues) that this molecule may reverse oxidative damage to DNA [83,84].

### 3.3. Anti-Inflammatory Potential of Melatonin

It was observed that melatonin exerts a therapeutic effect in acute and chronic inflammatory diseases. Esalatmanesh et al. found that therapy with melatonin decreased clinical signs of rheumatoid arthritis measured with the DAS-28 scale (although without a significant difference when compared to a placebo) [85]. Chojnacki et al. described a positive effect of adjuvant therapy with melatonin in patients with ulcerative colitis [86]. Sánchez-López et al. reported decreases in the concentrations of pro-inflammatory cytokines in patients suffering from relapsing–remitting multiple sclerosis [87]. Patients with chronic obstructive pulmonary disease treated with melatonin experienced dyspnea relief compared to a placebo group [88]. Therapy with melatonin led to reductions in the concentrations of pro-inflammatory cytokines and chemokines in septic patients [89].

The mechanism of the potential anti-inflammatory effect of melatonin was studied in animal and in vitro models. The most common effect of melatonin is a reduction in the release of pro-inflammatory cytokines. In an animal model of H1N1 virus infection, Huo et al. found that therapy with melatonin may suppress the migration of neutrophils and mast cells towards infected tissue and the production of pro-inflammatory cytokines by these cells [90]. In an LPS-evoked model of glial activation that resembles neuroinflammatory processes following ischemic brain injury, melatonin suppressed glial activation and the production of TNF-alpha, Il-1b, and Il-6 [91]. It was also proven that melatonin is capable of diminishing pyroptotic processes in ischemic brain injury [92]. This is probably due to the inhibition of signaling pathways dependent on sirtuin 3 and nuclear factor kB (NF-kB) [66,93]. This inhibition prevents the overexpression of inflammasome NLRP3 provoked by pro-inflammatory cytokines [94].

Thanks to its ability to diminish the concentrations of pro-inflammatory cytokines and suppress the activity of an inflammasome, melatonin is a promising agent that may exert a neuroprotective effect in secondary TBI resulting from neuroinflammation and other factors.

## 4. Role of Melatonin in TBI

### 4.1. Antioxidant Action of Melatonin in TBI

The very first event in the pathological cascade of TBI is the physical deformation of neural cells resulting from the action of the external force. Neurons (especially their axons) are strained, flexed, or twisted with a rapid change in the three-dimensional structure of the neurolemma [15]. This change impacts the properties of transmembrane ion channels, increasing their number and permeability, leading to a significant influx of ions (mainly calcium ions) into the cells. This surge in the cytosolic concentration of calcium ions has many consequences, with one of them being the uncoupling of the mitochondrial electron transport chain, which ultimately leads to the emergence of vast amounts of free radicals and the development of oxidative stress [95]. Melatonin acts at the very beginning of this process [96]. While trauma causes an increase in the number of transmembrane TRPM2 channels, which open to let calcium ions inside the cells, melatonin can inhibit the expression of these channels and reduce their activity [97]. This prevents mitochondrial dysfunction and the generation of oxidative stress [98]. It is speculated that pretreatment with melatonin and sustaining a high concentration may protect neural cells from the consequences of trauma by eliminating one of the crucial initial causes of secondary brain injury: the influx of ions into cells, which leads to subsequent oxidative brain damage and cell death.

A prerequisite of the protective mechanism described above is the presence of melatonin in the extra- and intracellular environments before the trauma, which is not achievable without melatonin pretreatment. Still, introducing melatonin during an ongoing oxidative brain damage cascade may limit the consequences of trauma. Melatonin has the potential to increase the expression of Nrf-2, which is the key factor that regulates the expression of antioxidative enzymes. Nrf-2 is physiologically activated during oxidative stress, penetrates the nucleus, joins an antioxidant response element (ARE), and induces transcription of genes of antioxidant enzymes, e.g., superoxide dismutase (SOD) [99,100,101,102]. It was also shown in an animal model of TBI that melatonin can facilitate Nrf-2’s migration towards the nucleus. Through this mechanism, the presence of melatonin may increase the activity of SOD and other enzymes capable of reducing superoxide particles [103]. Moreover, it was also shown that stimulating melatonin receptors in the central nervous system exerts an antioxidant and neuroprotective effect. Wang et al. observed that treatment with ramelteon, an agonist of the melatonin receptor, led to heightened concentrations of Nrf-2 in the nuclei of neurons, enhanced the expression of SOD, and prevented neuronal degeneration [104].

Limiting calcium-dependent mitochondrial lesions and activating antioxidant enzymes with melatonin reduces the intensity of the peroxidation of lipids present in cellular membranes. Through this mechanism, melatonin is able to stop the vicious cycle of mitochondrial dysfunction (lesioned mitochondrial membranes produce free electrons that are captured by oxygen atoms to become free radicals, which peroxidate the lipids of mitochondrial membranes, leading to further lesions). Moreover, a melatonin-dependent reduction in oxidative attacks against lipids of the neurolemma may limit the intracellular influx of ions (stopping another vicious cycle of secondary injury) and water. Through this process, the use of melatonin prevents brain edema [105]. Protecting cellular membranes increases the tightness of the BBB, protecting the nervous system from an inflow of water and pro-inflammatory molecules as well as infiltration by immunological cells [106]. Thus, the antioxidant action of melatonin reduces injury to cellular membranes and protects the brain from injury at multiple levels: it protects the brain from apoptosis at the cellular level, from neuroinflammation at the tissue level, and from edema at the organ level [107,108].

### 4.2. Anti-Inflammatory Effects of Melatonin in TBI

TBI initiated by an external force triggers a cascade of inflammatory processes that culminate in cell death. This cascade begins with the activation of immunocompetent cells within the central nervous system (CNS), most notably astrocytes, which subsequently produce pro-inflammatory cytokines, chemokines, and matrix metalloproteinases [35]. These inflammatory mediators alter neuronal cell metabolism towards pro-apoptotic pathways via inflammasome activation, concurrently increasing BBB permeability [38]. The resulting chemoattraction promotes infiltration by systemic immune cells, including leukocytes, monocytes, and macrophages, into the CNS, where they amplify the inflammatory response and further damage neural tissues through cytotoxic and phagocytic mechanisms [40]. Additionally, B lymphocytes activated by antigen-presenting cells are recruited to the brain, where they may produce anti-neuronal antibodies, contributing to chronic autoimmune neuroinflammation [43].

Melatonin demonstrates efficacy in inhibiting this inflammatory cascade at multiple stages. First, melatonin prevents the pathological activation of astrocytes and their shift towards a pro-inflammatory phenotype [109]. Melatonin has a similar effect on microglial cells, preventing them from transforming into the pro-inflammatory M1 phenotype [110]. In animal models, melatonin therapy has been shown to inhibit the expression of pro-inflammatory cytokines (e.g., IL-1β, IL-6, and TNF-α), chemokines, and matrix metalloproteinases while simultaneously promoting the secretion of anti-inflammatory cytokines, such as IL-10 [95,108]. This shift towards an anti-inflammatory cytokine profile mitigates pyroptosis and inflammatory neural tissue damage. Furthermore, melatonin-induced reductions in pro-inflammatory cytokine production and the presence of melatonin itself inhibit the expression and activation of the NLRP-2 inflammasome, reducing the intensity of trauma-induced neuroinflammation [111].

Melatonin’s anti-inflammatory effects extend to the downregulation of matrix metalloproteinase production. It is noteworthy that these enzymes contribute to the degradation of tight junction proteins within the BBB, increasing its permeability. By enhancing BBB integrity, melatonin reduces the infiltration of immune cells into the CNS [112]. Additionally, this melatonin-mediated BBB stabilization minimizes the leakage of neural antigens into the systemic circulation, preventing further stimulation of antigen-presenting cells and B lymphocytes. As a result, melatonin therapy may reduce the production of anti-neuronal antibodies and the progression of autoimmune neuroinflammation [113,114,115].

The reduction in inflammatory activity associated with melatonin also inhibits the complement system, thereby decreasing the formation of membrane attack complexes, which provides an additional neuroprotective mechanism against secondary traumatic injury [42]. It has been shown that melatonin exhibits various anti-inflammatory actions, giving it potential as a therapeutic molecule in TBI.

### 4.3. Clinical Studies on Melatonin in TBI

Clinical data on the role of melatonin in TBI are scarce and ambiguous but encouraging. Seifman et al. analyzed the relationships between the concentrations of melatonin in cerebrospinal fluid (CSF) and serum and measures of oxidative stress, radiological results, and clinical outcomes in TBI patients. The authors found that there was a significant increase in the CSF concentration of melatonin immediately after trauma. This increase was correlated with the CSF concentration of isoprostan—a marker of lipid peroxidation. Patients with radiological signs of diffuse axonal injury had lower levels of melatonin in their CSF. The serum concentration of melatonin was positively correlated with good clinical outcomes. The authors concluded that the observed increase in melatonin was a response to oxidative stress within the central nervous system (leading to the increased level of isoprostan), which suggests that melatonin was secreted in larger amounts due to its involvement in antioxidant activity. The antioxidant and anti-inflammatory properties of melatonin may explain the negative correlations between its concentration and signs of diffuse injury [116]. This study suggested a protective role of melatonin in TBI. In this context, the findings of two studies by Lorente et al., where a higher serum concentration of melatonin was positively correlated with mortality in the TBI population, may seem surprising [117,118]. The following line of reasoning was used to explain this finding: the more severe the TBI, the more intensive the oxidative stress and the higher the demand for endogenous melatonin. This is why the patients with the most severe TBIs, which led to their deaths, had higher serum concentrations of melatonin that were too low to control secondary brain injuries. This conclusion opens a discussion of whether using exogenous melatonin would benefit these patients. A beneficial effect of melatonin can be hypothesized based on a description of two cases of traumatic comas where improvement was paralleled by normalization of circadian rhythmicity (an effect of intensive use of melatonin), as published by Gobert et al. [119].

Barlow et al. performed a placebo-controlled clinical trial with melatonin usage (dosed at 3 or 10 mg per day) in children with postconcussive symptoms. Although their symptoms improved with time, there were no significant differences between the treatment and control groups [120]. Meanwhile, therapy with melatonin (3 mg or 10 mg daily) significantly improved sleep in children with postconcussive syndrome in a placebo-controlled study [121]. Reductions in sleep-related problems and increases in sleep duration and effectiveness were significantly larger in the treatment group than in the placebo group. Treatment with melatonin in this patient population led to noticeable changes in functional connectivity within brain regions, as observed in an MRI study [122]. Melatonin can also improve sleep in adult patients after TBI. Grima et al. performed a randomized, double-blind, placebo-controlled crossover study with melatonin (at a dose of 2 mg per day). Treatment with melatonin led to a significant improvement in sleep quality measured with the Pittsburgh Sleep Quality Index. The authors also observed an increase in sleep efficiency and better scores in some domains of quality of life resulting from melatonin intake [123]. Further analysis of data from that study revealed that more severe sleep disturbances were associated with more pronounced improvements [124].

Clinical studies focusing on melatonin usage in the acute and subacute phases of TBI are still lacking, and this constitutes an important knowledge gap. This, in turn, opens the possibility of planning prospective interventional studies with melatonin in the future.

## 5. Summary and Future Directions

TBI, especially its secondary and chronic phases, results from oxidative stress and inflammatory reactions rather than the direct effect of an external force. It is of special note that the primary effect (primary brain injury) is difficult to control clinically, and therapeutic interventions may inhibit processes that lead to secondary injury. Melatonin, with its antioxidative and anti-inflammatory profile, is still a promising therapeutic molecule. Data from in vitro studies and animal models are encouraging. Information emerging from human studies seems to confirm the potential for melatonin to alleviate tissue injuries and the clinical consequences of TBI. The next step in exploring melatonin usage is to plan prospective clinical trials assessing the benefits of using this neurohormone in TBI. Such trials, especially those with end points based on cognitive and behavioral symptoms, should focus on selecting appropriate patients and optimizing the timing and dosing of melatonin-based interventions.

## Data Availability

Not applicable.
